# Statistical Analysis of NMR Metabolic Fingerprints: Established Methods and Recent Advances

**DOI:** 10.3390/metabo8030047

**Published:** 2018-08-28

**Authors:** Helena U. Zacharias, Michael Altenbuchinger, Wolfram Gronwald

**Affiliations:** 1 Institute of Computational Biology, Helmholtz Zentrum München, Ingolstädter Landstraße 1, 85764 Neuherberg, Germany; helena.zacharias@helmholtz-muenchen.de; 2 Statistical Bioinformatics, Institute of Functional Genomics, University of Regensburg, Am Biopark 9, 93053 Regensburg, Germany; michael.altenbuchinger@ukr.de; 3 Institute of Functional Genomics, University of Regensburg, Am Biopark 9, 93053 Regensburg, Germany

**Keywords:** data normalization, data scaling, zero-sum, metabolic fingerprinting, NMR, statistical data analysis

## Abstract

In this review, we summarize established and recent bioinformatic and statistical methods for the analysis of NMR-based metabolomics. Data analysis of NMR metabolic fingerprints exhibits several challenges, including unwanted biases, high dimensionality, and typically low sample numbers. Common analysis tasks comprise the identification of differential metabolites and the classification of specimens. However, analysis results strongly depend on the preprocessing of the data, and there is no consensus yet on how to remove unwanted biases and experimental variance prior to statistical analysis. Here, we first review established and new preprocessing protocols and illustrate their pros and cons, including different data normalizations and transformations. Second, we give a brief overview of state-of-the-art statistical analysis in NMR-based metabolomics. Finally, we discuss a recent development in statistical data analysis, where data normalization becomes obsolete. This method, called zero-sum regression, builds metabolite signatures whose estimation as well as predictions are independent of prior normalization.

## 1. Introduction

Metabolomics is defined as the comprehensive study of small organic compounds, so-called metabolites, in a biological specimen, e.g., a cell, an organ, or a whole organism. Particular focus is placed on the identification of metabolites that characterize specific phenotypes. These metabolic biomarkers can facilitate new insights into pathomechanisms of diseases, as well as offer efficient diagnostic tools and possible targets for patient treatment.

Typical metabolites are amino acids, sugars, organic acids, bases, lipids, vitamins, and various conjugates of absorbed substances of exogenous origin. Metabolomics finds widespread application, including such diverse topics as the screening of milk of dairy cows [1] or the investigation of acute kidney injury following heart surgery [2,3].

Metabolomic investigations are mainly conducted employing hyphenated mass spectrometry or nuclear magnetic resonance (NMR) spectroscopy. Here, we will focus on the application of solution NMR spectroscopy to biological fluids as well as tissue and cell extracts in an academic setting, although many of the described approaches are not limited to these examples.

In order to extract meaningful information from NMR metabolic fingerprints, numerous statistical data analysis methods are applied. Routinely, the significance of differential metabolite intensities is assessed by hypothesis testing. Unsupervised machine learning methods are applied to unravel structure in the data, and supervised machine learning methods try to separate predefined groups of specimens.

Researchers in NMR-based metabolomics are confronted with a vast amount of different methods, making it challenging to decide on one or the other. The intention of this review is twofold. First, we want to provide a brief overview of available data processing and analysis techniques, without the intention of being complete. Second, we want to point the reader towards potential issues. Data analysis is not unique. Different methods yield different results, and even final conclusions can be altered. This particularly concerns the preprocessing of data. We will review an example where prior data normalization substantially influences the downstream analysis and its interpretation. Finally, we will describe a recent development where parts of the data preprocessing no longer impact downstream analysis.

## 2. Preprocessing

### 2.1. Data Extraction

Routine statistical analysis requires predefined molecular features. These features are either defined in a targeted manner, where they constitute preselected, often absolutely quantified metabolites, or in an untargeted manner, where they comprise the whole spectral region. The latter approach does not require the identification of metabolites prior to feature extraction, and is recommended in the context of exploratory phenotype analysis.

Different methods for data extraction in NMR-based metabolomics are available. In general, NMR signal positions can vary across specimens due to differences in pH, ionic strength, or measurement temperature. A widely used and robust method to at least partially compensate for these effects is spectral binning. A simple but efficient strategy implies the splitting of the whole spectral region into equally spaced buckets/bins. Data points inside every bucket are summed up or integrated. The whole dataset is then represented as a matrix of bucket integrals, where the rows correspond to individual specimens and the columns correspond to individual bins, respectively. Other schemes such as adaptive binning [4], spectral alignment [5,6], and combinations thereof have been shown to be superior to equidistant binning [4,5,7,8,9].

### 2.2. Normalization

Metabolomic datasets are prone to unwanted technical and/or biological variances and biases. Technical variances can result from differences in sample collection, storage, and preparation, as well as spectrometer performance across the set of investigated specimens. Unintended biological variances can arise due to numerous reasons. The most prominent, in the case of urine metabolomics, are dilutions of metabolite concentrations due to the varying fluid intake of study participants. Urine specimen dilutions can further vary due to drugs, toxins, disease status, respiration, defecation, perspiration, and patient treatment [10,11]. Other reasons for unintended biological variances may be differences in the available sample volumes due to unequal numbers of cells in the case of cell-line extracts, varying tissue volumes/masses, or varying biofluid volumes across the investigated cohort.

To minimize the undesired technical and biological variance across specimens is the goal of data normalization in metabolomics. As stated by Craig et al. (2006) [10], it can be considered as a row operation to remove unwanted sample-to-sample variations. Numerous normalization methods have been suggested during the past years, and we will briefly introduce the most prominent strategies for NMR-based metabolomics.

**Figure 1:**
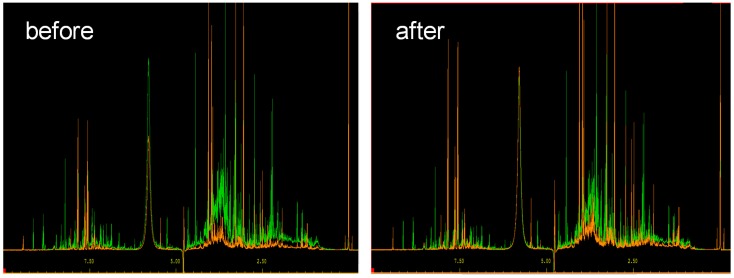
Normalization of two different urine spectra with respect to creatinine. (**Left**) Before normalization and (**right**) after normalization.

A normalization of each metabolic fingerprint to a specific “housekeeping” metabolite, e.g., creatinine, is a common approach to remove data variances due to differences in overall urine concentrations [10,12], as exemplified in Figure 1. Here, creatinine clearance is assumed to be constant and used as a proxy for renal function [10]. However, this normalization strategy cannot be recommended in general [12]. It assumes the absence of interindividual differences in the production and renal excretion of creatinine [13]. However, creatinine production and excretion depend on the sex, age, muscle mass, diet, and pregnancy, as well as renal pathology of the examined individual [14,15].

Another commonly applied method to reduce unwanted sample-to-sample variation is the normalization of every spectrum to a total sum of one, the so-called total spectral intensity/area normalization. Here, each metabolic feature is divided by the total sum of all of the spectral features. It assumes that only a relatively small amount of metabolites is regulated in approximately equal shares up and down, while all others remain constant. However, this prerequisite is often not fulfilled, especially in the case of kidney diseases where, e.g., higher overall blood metabolite levels are observed in diseased than in healthy patients [3].

Probabilistic quotient normalization (PQN) assumes that biologically interesting concentration changes only affect parts of the NMR spectrum, whereas different specimen dilutions influence all of the metabolite signals simultaneously [16]. For PQN, each spectrum first is normalized to the total spectral intensity, and multiplied by 100. Subsequently, a reference spectrum, e.g., the median spectrum over all of the spectra of a cohort is derived, and the quotient of all of the variables of the investigated spectra and the reference spectrum is calculated. In the next step, the median of these quotients across all of the metabolic features is computed, and finally, each metabolic feature of the spectra is divided by this median. Again, this normalization method is not applicable if the underlying assumptions are not fulfilled.

If only technical variances due to differences in spectrometer performance need to be addressed, a normalization to the NMR reference substance is recommended [3].

We systematically compared established and advanced normalization methods for urinary metabolomic NMR datasets [17]. Here, quantile [18], variance stabilization [19] and cubic spline [20] normalization performed best with respect to sample classification, bias reduction, and the detection of correct fold changes. However, these methods all assume that only a relatively small proportion of the metabolites is different between the investigated groups, and therefore, the average total spectral areas are assumed to be similar across specimens and groups [21]. If this assumption is not fulfilled, Hochrein et al. 2005 [21] suggest to learn the normalization parameters on a subset of non-regulated features only. Additionally, it is important to note that all of the different normalization strategies mentioned here impact the following analysis steps such as screening for differential metabolites or multivariate metabolic signatures [22,23,24,25], as we will discuss in more detail in the following sections.

### 2.3. Additional Data Transformation

In addition to normalization, further data transformations might be necessary. Many statistical methods assume variables that are distributed multivariate normal and have constant variance. Binned NMR intensities frequently exhibit skewed distributions across specimens, i.e., the data are heteroscedastic. The most prominent method to achieve approximately normal distributed variables and equal variance is the logarithmic transformation, which was suggested in the context of NMR-based metabolomics, e.g., by Viant et al. (2005) [26]. A mathematically more evolved method, which requires the estimation of an additional parameter, is a variance stabilizing transformation (VST) [27]. VST in combination with normalization is variance stabilization normalization (VSN) [19]. This method was systematically evaluated in Kohl et al. (2012) [17], where it was among the best preprocessing strategies and particularly performed well for both classification and bias removal. Other data transformations that do not account for heteroscedasticity but which correct the variance of variables are, e.g., Pareto [28] and autoscaling [29]. Both methods rescale metabolite features; thus, they are column operations in contrast to normalization, which is a row operation according to Craig et al. (2006) [10]. A detailed discussion of these strategies is beyond the scope of this article and we refer the interested reader for example to Craig et al. (2006) [10], Kohl et al. (2012) [17], Gromski et al. (2015) [23], van den Berg et al. (2006) [30], and Emwas et al. (2018) [31] for more details.

## 3. Statistical Data Analysis Strategies

### 3.1. Unsupervised Machine Learning Methods

In unsupervised machine learning, no information about underlying groups is used. Therefore, the group separations that are observed are purely data-driven. Unsupervised algorithms are often employed to check for group separation prior to the classification of data or in cases where too few samples are available for classification with rigid cross-validation.

One of the most prominent unsupervised methods in the metabolomics community is principal component analysis (PCA). PCA is a dimension reduction approach where new coordinate axes in the directions of maximal variances are drawn. The maximum variance is not necessarily equal to the intended biological variance, i.e., the metabolic differences between different phenotypes, but might also arise from for example batch effects, which had not been successfully removed by data normalization. PCA enables the easy visualization of high-dimensional data. Closely related to PCA is independent component analysis (ICA), which has been shown to provide good results for metabolic data [32,33].

Other used methods include clustering approaches such as hierarchical clustering [34], non-hierarchical clustering employing the k-means method [35], and clustering by affinity propagation [36]. Self-organizing maps are a widely used method for two-dimensional data visualization [37]. For more details about unsupervised machine learning in the context of NMR-based metabolomics, we refer the interested reader for example to Zacharias et al. (2013) [38].

### 3.2. Hypothesis Testing

Hypothesis tests are of central importance in metabolomics data analysis. They are used to identify differentially regulated metabolites. For instance, a standard application is to screen for metabolites that serve as biomarkers of a certain disease. Here, metabolite intensities are compared between healthy and diseased individuals. Routinely, in the case of normally distributed data, this is done by applying a Student’s *t*-test or, if there are more than two conditions to be compared, an analysis of variance (ANOVA).

Since common metabolic studies comprise a large number of metabolic features, significance levels or *p*-values need to be corrected for multiple hypothesis testing. Prominent methods are controlling the false discovery rate according to Benjamini and Hochberg [39] and controlling the familywise error rate according to Bonferroni [40].

**Figure 2:**
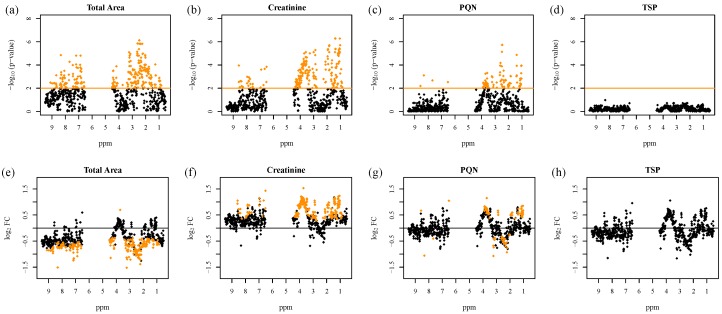
Test for differentially regulated metabolites in 1D ^1^H urinary nuclear magnetic resonance (NMR) fingerprints between acute kidney injury (AKI) and healthy patients with respect to different normalization strategies. –Log_10_(*p*-values) of moderated *t*-test analysis are shown after preprocessing with four different normalization methods: scaling to (**a**) equal total spectral area, (**b**) scaling to creatinine, (**c**) probabilistic quotient normalization (PQN), and (**d**) scaling to the internal reference TSP, plotted versus the ppm regions of the corresponding NMR buckets (upper panels). The significance level for Benjamini–Hochberg (B/H) adjusted *p*-values below 0.01, corresponding to a false discovery rate (FDR) below 1%, is marked by an orange line, and the significant NMR features are indicated as orange diamonds. The corresponding log_2_ fold changes (log_2_ FC) plotted versus the ppm regions are shown in the lower panels (**e**–**h**). Since log_2_ FCs were calculated as AKI minus non-AKI, positive log_2_ FCs correspond to higher values in AKI than in non-AKI samples. Figure adapted from Zacharias et al. (2017) [22].

However, the results of univariate data analysis exhibit a distinct dependency on the a priori chosen normalization method, as investigated by Zacharias et al. (2017) [22]. Figure 2 illustrates these observations for a *t*-test analysis of urinary NMR fingerprints of acute kidney injury (AKI) versus healthy patients.

The number as well as the identity of statistically significant NMR buckets strongly depends on the employed normalization strategy. This finding points to an inherent problem of standard statistical data analysis in metabolomics studies: the respective results are always dependent on the often arbitrarily chosen normalization strategy, and findings can probably only be reproduced if the initial choice of normalization is used.

### 3.3. Supervised Machine Learning Methods

The classification of an unknown sample into two or more known phenotypic classes (e.g., healthy and diseased) is a common task for which techniques from machine learning are used.

Popular machine learning methods in omics science are partial least squares discriminant analysis (PLS-DA) [41], orthogonal projection to latent structures discriminant analysis (OPLS-DA) [42], random forest (RF) [43], support vector machine (SVM) [44], as well as least-absolute shrinkage and selection operator (LASSO) [45], ridge [46], and elastic net regression [47].

In metabolomic data analysis, PLS-DA and OPLS-DA are most widely used. RF and SVM are less frequently applied, but are well established, for example, in gene expression analysis. Hochrein et al. (2012) [48] showed that RFs are particularly well suited for the analysis of high-dimensional NMR data with regard to prediction accuracy [48]. Elastic net, or its special cases ridge and LASSO regression, are also rather unpopular in metabolomics data analysis. However, they are very popular in the machine learning community, and exhibit excellent performance also in NMR metabolomics [48]. All methods have their pros and cons, and several comprehensive comparisons are available, e.g., Hochrein et al. (2012), [48], Gromski et al. (2015) [49], Ren et al. (2015) [50], and Cuperlovic-Culf et al. (2018) [51].

A particular challenge in supervised machine learning is the high-dimensionality of the data. Usually, many more metabolic features are assessed than specimens are available. As a consequence, high performance on the training data does not necessarily imply high performance on the test data, which is commonly known as the problem of over-fitting. Therefore, results need to be validated by bootstrapping, cross-validation, or in the best case, on independent validation data. Although state-of-the-art machine learning methods are designed to control over-fitting, such as LASSO/ridge regression, SVMs, and random forests, their performance remains to be validated.

**Figure 3:**
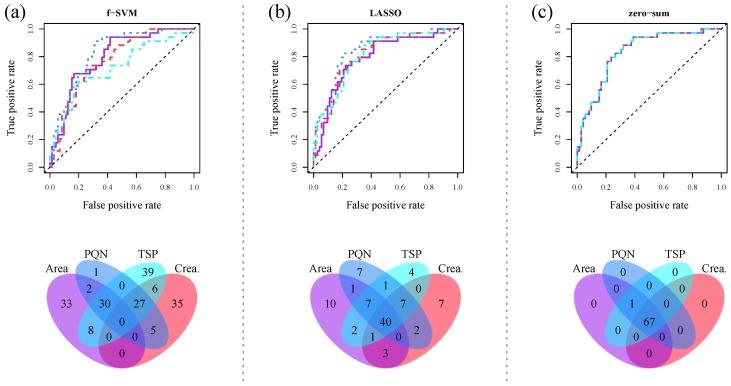
Receiver operating characteristic (ROC) curves as well as Venn diagrams of selected classification features for the discrimination of AKI from non-AKI patients based on urinary 1D ^1^H NMR fingerprints. Four different normalization strategies were employed: scaling to total spectral area (violet solid line), scaling to creatinine (red dashed line), probabilistic quotient normalization (PQN) (blue dotted line), and scaling to the internal reference TSP (cyan dashed–dotted line). Common classification approaches such as (**a**) support vector machine (SVM) in combination with t-test based feature filtering, and (**b**) least-absolute shrinkage and selection operator (LASSO) regression show a clear dependence on the chosen normalization strategy, whereas (**c**) zero-sum regression is completely independent thereof. Figure adapted from Zacharias et al. (2017) [22].

As previously, data preprocessing is essential for the application of supervised machine learning methods. As shown in Zacharias et al. (2017) [22] and in Gromski et al. (2015) [23], data normalization impacts the performance of supervised machine learning methods. We exemplarily illustrate the effect of normalization on classification performance and feature selection for urinary NMR fingerprints in Figure 3a,b.

Both the performance and the derived metabolic signatures for (a) a SVM in combination with t-test based feature filtering, and (b) a LASSO classification strongly depend on the a priori chosen normalization strategy. For a corresponding figure for sparse PLS-DA, we refer the interested reader to Zacharias et al. (2017) [22]. Consequently, the reproducibility of metabolic studies is dependent on the normalization and classification methods employed. Accordingly, reproducible classification results are only achievable when the exact same preprocessing protocols are used. This limits the applicability of metabolic signatures derived by standard statistical analysis approaches.

## 4. Zero-Sum Regression

To overcome these limits of traditional biomarker signatures, zero-sum regression [52,53], which has recently been demonstrated to be invariant under any normalization of data [53], has been extended to logistic zero-sum regression [22]. In contrast to commonly used approaches, logistic zero-sum regression always selects the same set of biomarkers for sample classification, regardless of the chosen normalization method. Therefore, prior data normalization may be omitted completely.

In brief, it is based on the following concept: We start with the binned fingerprinting data *x_ij_*, where *x_ij_* is the logarithm of the intensity of bin *j* ∈ {1,…,*p*} in spectrum *i* ∈ {1,…,*N*}, and *y_i_* is the corresponding (clinical) response of patient *i*. In standard regression analysis, prior data normalization to a common unit such as the total spectral area is required. As the data are on a logarithmic scale, normalization to a common unit becomes a shifting of the binned value *x_ij_* by some spectrum-specific value *γ_i_*. Therefore, in the case of normalized data, the regression equation reads:

(1) $$y_{i}=\beta_{0}+\overset{p}{\underset{j=1\;}{\sum }}\beta_{j}\left(x_{ij}+\gamma_{i}\right)+\epsilon_{i}$$

Equation (1) becomes independent of the normalization factor *γ_i_* if and only if the regression coefficients *β_j_* sum up to zero, i.e.:

(2) $$\overset{p}{\underset{j=1\;}{\sum }}\beta_{j}=0.$$

As a consequence, the additional constraint that all regression coefficients have to sum up to zero is set in zero-sum regression. Zacharias et al. (2017) [22] showed for two metabolomic datasets that the obtained biomarker signatures were indeed independent of any prior data normalization. Figure 3c illustrates these results for a urinary 1D ^1^H NMR metabolic dataset.

## 5. Available Software for Metabolomics Data Preprocessing and Statistical Analysis

The statistical programming environment *R* [54] provides a convenient way of normalizing and transforming datasets, as well as performing subsequent data analysis. Other common tools to perform these tasks or parts thereof include, for example, the numerical programming environment MATLAB (The MathWorks Inc., Natick, MA, USA), the online server MetaboAnalyst [55], MVAPACK [56], Workflow4Metabolomics [57], and the data analysis software SIMCA (Umetrics, Umeå, Sweden). Most recently, NormalizeMets has been proposed for the comparative evaluation of normalization methods in metabolomics studies [58]. Another web tool, called MetaPre, offers the possibility of evaluating the normalization performance of, in total, 16 different normalization methods [59]. Logistic as well as linear zero-sum regression are available as an *R* package and as high-performance computing software at https://github.com/rehbergT/zeroSum.

## 6. Conclusions

In this review, we focused on the statistical data analysis of NMR-derived metabolic fingerprints. Special emphasis was given to the issue of data normalization and its impact on downstream analysis and result interpretation. In this context, we focused on the novel logistic zero-sum regression method that is independent of prior data normalization, and therefore has the potential to greatly enhance the reproducibility of biomarker studies.
